# Hemorrhagic Pericardial Effusion Leading to Cardiac Tamponade: A Rare Initial Presentation of Adenocarcinoma of the Lung

**DOI:** 10.7759/cureus.11411

**Published:** 2020-11-10

**Authors:** Maham A Mehmood, Monica Bapna, Ayesha Siddiqa, Asim Haider, Muhammad Saad

**Affiliations:** 1 Internal Medicine, BronxCare Hospital Center, Bronx, USA; 2 Internal Medicine, King Edward Medical University, Lahore, PAK; 3 Cardiology, BronxCare Hospital Center, Bronx, USA

**Keywords:** pericardial effusion, cardiac tamponade, metastatic, chemotherapy-related toxicity, metastatic lung

## Abstract

Malignancy associated pericardial effusion is a serious condition and testifies to poor prognosis. Cardiac tamponade can be the first presentation of underlying adenocarcinoma of the lung. We present a 78-year-old female with no known history of any malignancy, who presented with symptoms of abdominal and respiratory pathology. The physical exam findings were significant for a possible cardiac tamponade. Computed tomography (CT) of the abdomen and chest confirmed moderate bilateral pleural effusions, large pericardial effusion, left upper lobe mass, possible lymphangitic spread of carcinoma in the left lung, and adenopathy in the mediastinum. The echocardiography findings further confirmed tamponade. Cardiology and pulmonary medicine were taken on board for a possible malignancy associated pleural effusion leading to cardiac tamponade. Pericardial fluid analysis showed atypical cells suggestive of malignancy. Transbronchial biopsy confirmed moderately differentiated invasive adenocarcinoma. Positron emission tomography (PET) scan revealed metastatic spread to the mediastinum and right hilum with possible pleural metastatic disease seen posteriorly in the left hemithorax. The patient was discharged home with oncology follow up for chemotherapy.

## Introduction

Adenocarcinoma is the most common type of lung cancer, accounting for one-half of lung cancer cases. A hemorrhagic pericardial effusion, leading to cardiac tamponade, as the first manifestation of an undiagnosed malignancy, is an uncommon and fatal presentation. The sites of distant metastasis are bone, brain, liver, and adrenal glands [1]. Cardiac metastasis is a harbinger of poor prognosis [2-3]. Our case emphasizes the importance of timely intervention in malignancy associated pleural effusions leading to tamponade. Prompt recognition can be lifesaving and provides symptomatic relief, and helps in the diagnosis of the etiology.

## Case presentation

**Figure 1:**
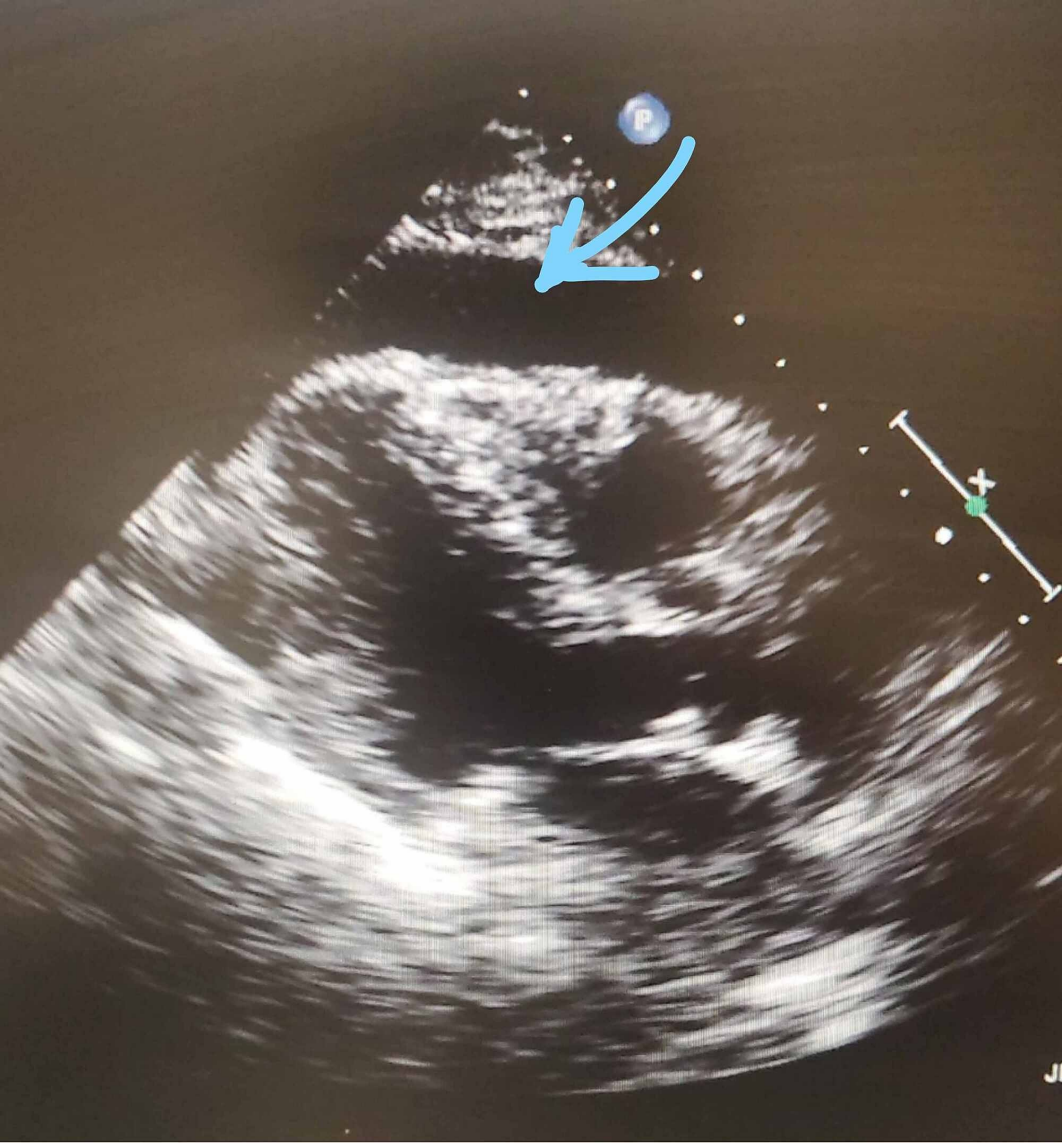
Echo showing parasternal long axis view. Pericardial effusion (blue arrow)

Our patient is a 78-year-old female with a medical history significant for Charcot-Marie-Tooth disease, vertigo, hypertension, hyperlipidemia, depression, and Hashimoto's thyroiditis, who presented to the hospital for abdominal pain, constipation, shortness of breath, and 10 lbs weight loss for the last few weeks. In the emergency room, the vitals were temperature of 98.3 F, heart rate 100 beats per minute, respiratory rate 18 breaths/minute, Blood pressure 120/70 mm of Hg, and oxygen saturation of 98% on room air. The physical examination was unremarkable at admission. Her hemoglobin was 10.9 g/dl, white blood cell 5.8 k/ul, platelet count of 270 k/ul, alkaline phosphatase 214 u/l, aspartate aminotransferase 347 u/L, alanine aminotransferase 409 u/L, troponin 31 ng/L, 34 ng/L, and pro-B-type natriuretic peptide (pro-BNP) 265 pg/ml. The patient underwent computed tomography (CT) of the abdomen and pelvis, which showed moderate bilateral pleural effusions, a large pericardial effusion, and the liver's periportal edema. There is a gallbladder wall edema correlating to elevated right heart pressures. The patient also underwent CT of the chest, which was suspicious for the left upper lobe's lung malignancy, pleural effusions, atelectasis, a large pericardial effusion, and pathological adenopathy of the mediastinum. The echocardiographic study was significant for massive pericardial effusion with right ventricular diastolic collapse indicating cardiac tamponade (Figure 1).

**Figure 2:**
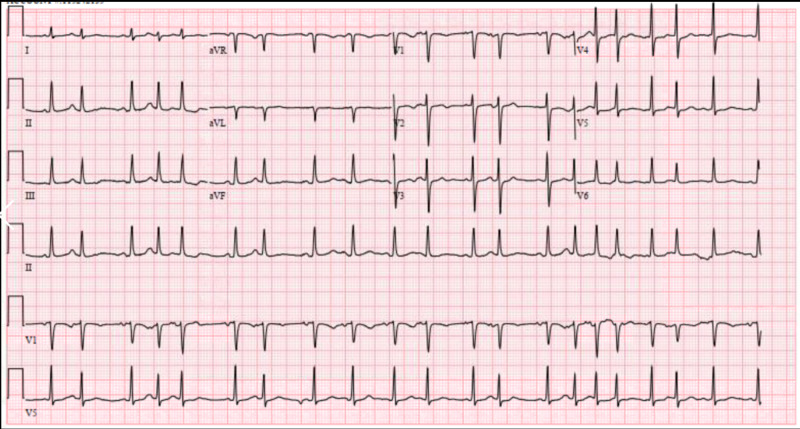
Figure 1: EKG Showing atrial fibrillation and electrical alternans

Pulmonary medicine was consulted for the left upper lobe lung mass and bilateral pleural effusion. The patient was evaluated by cardiology, and examination was significant for pulsus paradoxus of 12-14 mm of Hg, Becks triad, electrical alternans, low voltage QRS, and Ewart sign (Figure 2).

**Figure 3:**
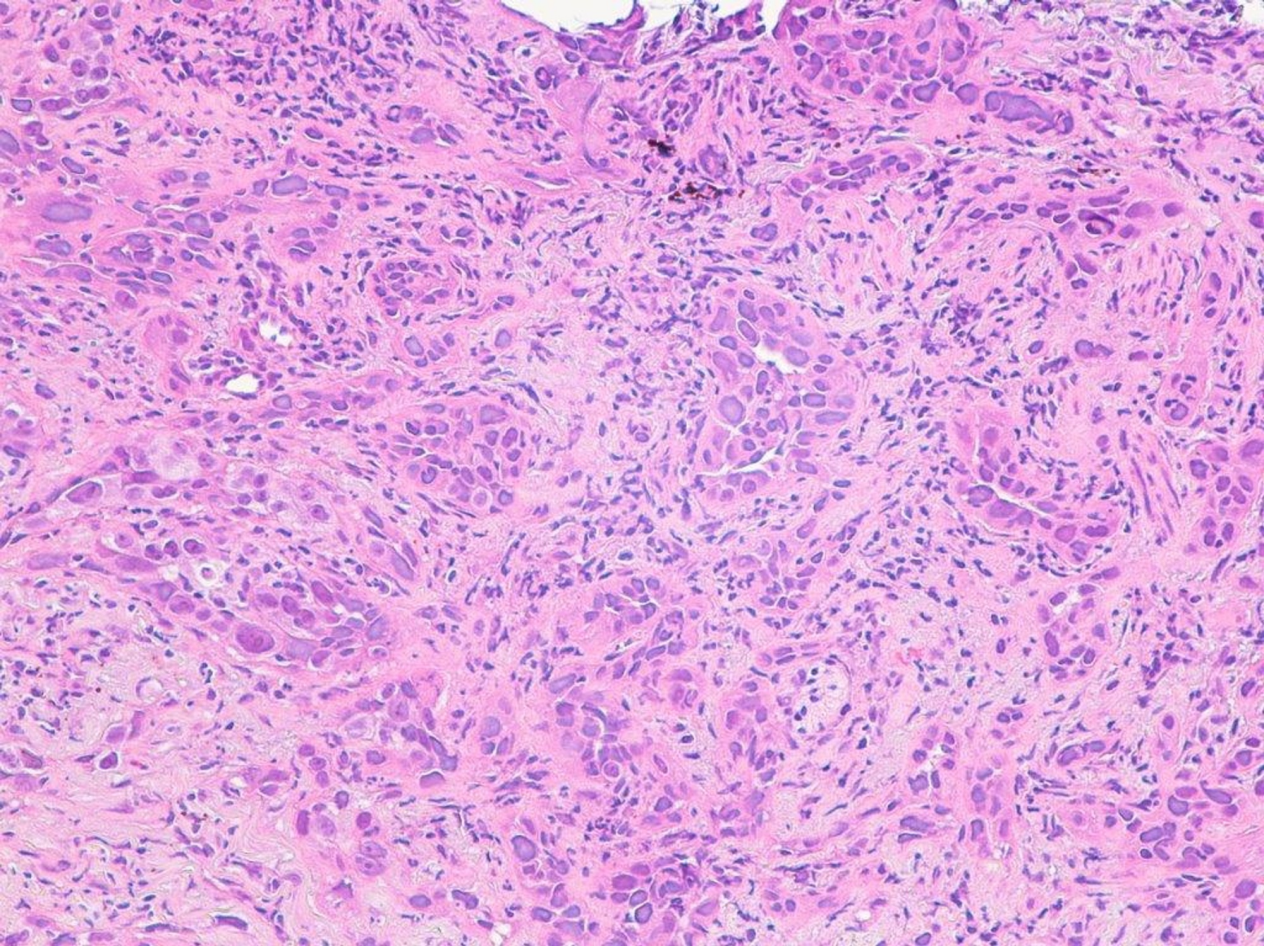
Lung transbronchial biopsy showing acinar pattern of adenocarcinoma, composed of glands lined by malignant cells with desmoplastic stroma (hematoxylin and eosin magnification x200)

The patient went to the critical care unit and underwent pericardiocentesis with drain placement. 1600 ml fluid was drained over two days, which was hemorrhagic with atypical cells suggestive of malignancy. The pericardial fluid was bloody, cloudy, white blood cell (WBC) 4700 cells/mm3 with 92% segmental count, and 29440000 red blood cell (RBC) cells/mm3. The patient developed paroxysmal atrial fibrillation; however, she was not a candidate for anticoagulation due to hemorrhagic pericardial tamponade. The repeat echocardiography showed resolved pericardial tamponade. The patient underwent bronchoscopy, and transbronchial biopsy showed invasive adenocarcinoma, which was moderately differentiated (Figure 3).

After 15 days, the patient underwent a positron emission tomography (PET) scan, which showed a left upper lobe primary tumor with evidence of metastatic spread to the mediastinum and right hilum with possible pleural metastatic disease seen posteriorly in the left hemithorax. The patient was diagnosed with stage IV adenocarcinoma of the lung and was discharged to follow up outpatient with oncology for chemotherapy. The patient did follow up and tolerated the chemotherapy without complication at the fourth month follow up.

## Discussion

The pericardium is the most frequent site of metastatic involvement in primary lung cancer as far as the heart is concerned. The pericardium's malignant association is detected by up to 20% of cancer cases in autopsy studies. 10%-25% of times, large pericardial effusions tend to be cancerous. The myocardium's direct involvement is much less frequent, either by metastatic or primary tumors [1]. The secondary metastatic disease coming from the lungs and breast account for nearly 30% of reported cases and are more frequent due to their anatomical vicinity. The non-small cell lung cancers harbor 5% of anaplastic lymphoma kinase (ALK) rearrangement that is particularly likely to involve the pericardium or present with pericardial effusion [2]. The other etiologies of malignant pericardial effusion are post-radiation therapy, chemotherapy-induced, infection in immunocompromised, autoimmune diseases, and idiopathic pericarditis [3]. Majority of studies have shown that the leading cause of tamponade is iatrogenic, followed by malignancy, acute myocardial infarction, and idiopathic diseases. 

The hemorrhagic and malignant pericardial effusion leading to cardiac tamponade is an uncommon presentation of lung cancer. It is encountered in about 2% of patients. Cardiac tamponade occurs when blood, fluid, or pus builds up in the space between the myocardium and the pericardium. Malignant effusions of the pericardium are hemorrhagic, accumulate rapidly, and large average aspirate ranges between 840 and 1200 ml [4]. The progression of pericardial effusions to cardiac tamponade depends on the rate and volume of fluid collection. As low as 150 ml of rapid fluid buildup can cause tamponade, whereas up to 2 L of slow collection may not lead to tamponade [5]. Cardiac tamponade can be acute (rapid accumulation of fluid, 100-200 ml fluid) caused by chest trauma leading to rupture of the heart's free wall, post-myocardial infarction, and aortic dissection. Subacute/chronic cardiac tamponade is a result of the slow accumulation of fluid (up to 2000 ml fluid) caused by neoplasia and tuberculosis. Cardiac tamponade is classified based on the distribution (loculated and circumferential), hemodynamic impact (effusive or constrictive), composition (exudative, transudative), blood, air, or gas from infections [4]. The classic signs and symptoms are exertional dyspnea progressing to orthopnea, chest discomfort, or pain. Other non-specific symptoms are related to local compression-like nausea(diaphragm),dysphagia(esophagus), hoarseness (recurrent laryngeal nerve), hiccups (phrenic nerve). Pericardial fluid's compressive effect can cause sinus tachycardia and hypotension leading to symptoms like fatigue, palpitations, and anorexia [6].

The clinical presentation, as well as imaging, can point towards the diagnosis. Abnormal EKGs are seen in 90% of patients; most changes were non-specific except electrical alternates in 5% of patients. The echocardiography findings of the thickened pericardium with evidence of right ventricular diastolic collapse are the gold standard for the diagnosis of cardiac tamponade. A chest X-ray shows an enlarged cardiac silhouette. CT is a beneficial imaging modality and helps in recognizing calcified structures, measuring pericardial thickness, and treatment planning of neoplastic effusions. The cardiac magnetic resonance (CMR) is superior to CT as it can differentiate high proteinaceous exudative fluid from the thickened pericardium [7]. PET/CT in patients with solid tumors and lymphoma may symbolize malignant pericardial involvement, which assists in diagnosis and prognosis [8].

Fraser hypothesis sheds light on tamponade's pathogenesis via retrograde lymphatic spread from malignant mediastinal lymph nodes, as seen in our patient. The obstruction of lymphatic and venous outflow from mediastinal lymph nodes to the epicardial lymphatic plexus by the tumor results in effusion [9]. It has recently been proven that mast cells are required for malignant effusions formation. They release tryptase AB1 and interleukin‐1β (IL‐1β) that induce pleural vasculature leakiness and trigger nuclear factor kappa B (NF-κB) activation, thereby fostering fluid accumulation and tumor growth. The lung's endobronchial squamous cell carcinoma typically presents as cardiac tamponade; however, adenocarcinomas of the lung, which is anatomically more distal to lung parenchyma have higher chances of lymphatic obstruction.

To date, there is no established agreement on the most relevant management of cardiac tamponade. The numerous management options used are pericardiocentesis, pericardiectomy, and the creation of a pericardial window. Pericardiocentesis is a safe procedure when performed under an echocardiogram. It provides paramount relief, is conceivably life-saving, and the fluid can be used for sampling [10]. The rare complications include coronary arteries rupture, pneumothorax, and arrhythmias [11]. Surgical drainage may be indicated in patients when pericardiocentesis fails. Peri-cardiectomy has become uncommon due to its high operative risks [12]. For recurrent malignant effusions, a pericardial window is created, where the pericardium is sutured to the left lung's pleura and left to drain cancer) are the most active chemotherapeutic agents. Tetracycline, as a sclerosing agent, has outstanding results but has side effects of arrhythmias, chest pain, and recurrent fever [12]. For radiosensitive tumors such as lymphomas and leukemias, radiation therapy gives good results but can cause pericarditis and myocarditis [13].

Twenty-eight day mortality in patients with malignant cardiac tamponade is 33%, with a mean survival of around 144 days. Malignant fatal cardiac tamponade accounts for up to 30% of cases seen in autopsy studies; however, they are less commonly identified during the lifetime. The most prevalent cause of death is cardiac or respiratory arrest. Our patient came with progressive shortness of breath and non-specific symptoms. The pericardial fluid was showing malignant cells, and a lung biopsy demonstrating adenocarcinoma of the lung with metastasis to the mediastinal lymph node. She has not developed the recurrence of pericardial effusion and has survived over the mean survival duration. She was offered palliative follow up, which she refused and continued her treatment and follow up.

## Conclusions

Cardiac tamponade is a life-threatening condition and a medical emergency. The prognosis depends on prompt recognition and management of the tamponade. Cardiac tamponade is a clinical diagnosis with an echocardiogram used for confirmation. Some features of echocardiography may precede the clinical signs. The progression of the disease relies on its etiology. Untreated cardiac tamponade is rapidly and universally fatal. One-year mortality was 76.5% in patients with underlying malignant disease vs. 13.3% in patients without malignancy. The median survival of 150 days was noted in patients with malignant disease. As physicians, one should maintain a high index of suspicion for the possibility of malignancy in patients presenting with findings consistent with cardiac tamponade and hemorrhagic pericardial effusion. Identifying this unique presentation needs timely and proper treatment. Excellent medical comprehension and a timely pericardiocentesis can be life-saving as well as diagnostic.

## References

[1] Maisch B, Ristic A, Pankuweit S (2010). Evaluation and management of pericardial effusion in patients with neoplastic disease. Prog Cardiovasc Dis.

[2] Doebele RC, Lu X, Sumey C (2012). Oncogene status predicts patterns of metastatic spread in treatment-naive nonsmall cell lung cancer. Cancer.

[3] Wilkes JD, Fidias P, Vaickus L, Perez RP (1995). Malignancy-related pericardial effusion. 127 cases from the roswell park cancer institute. Cancer.

[4] Lazaros G, Stefanadis C (2013). Malignant pericardial effusion: still a long way to Ithaca?. Cardiology.

[5] Caballero LM, Asensio JMN, Alonso-Gonzalez R, Romero JJG, Fernández RG, Fernández AMP (2006). Constrictive pericarditis as the first sign of lung cancer [Article in English, Spanish]. Arch Bronconeumol.

[6] Muir KW, Rodger JC (1994). Cardiac tamponade as the initial presentation of malignancy: is it as rare as previously supposed?. Postgrad Med J.

[7] Wang ZJ, Reddy GP, Gotway MB, Yeh BM, Hetts SW, Higgins CB (2003). CT and MR imaging of pericardial disease. RadioGraphics.

[8] De Wever W, Ceyssens S, Mortelmans L, Stroobants S, Marchal G, Bogaert J, Verschakelen JA (2007). Additional value of PET-CT in the staging of lung cancer: comparison with CT alone, PET alone and visual correlation of PET and CT. Eur Radiol.

[9] Fraser RS, Viloria JB, Wang NS (1980). Cardiac tamponade as a presentation of extracardiac malignancy. Cancer.

[10] Imazio M, Colopi M, De Ferrari GM (2020). Pericardial diseases in patients with cancer: contemporary prevalence, management and outcomes. Heart.

[11] Tsang TSM, Seward JB, Barnes ME, Bailey KR, Sinak LJ, Urban LH, Hayes SN (2000). Outcomes of primary and secondary treatment of pericardial effusion in patients with malignancy. Mayo Clin Proc.

[12] Virk SA, Chandrakumar D, Villanueva C, Wolfenden H, Liou K, Cao C (2015). Systematic review of percutaneous interventions for malignant pericardial effusion. Heart.

[13] Celerrnajer DS, Boyer MJ, Bailey BP, Tattersall MHN (1991). Pericardiocentesis for symptomatic malignant pericardial effusion: a study of 36 patients. Med J Aust.

